# Community structure of endophytic bacteria of *Sargassum thubergii* in the intertidal zone of Qingdao in China

**DOI:** 10.1186/s13568-024-01688-2

**Published:** 2024-04-13

**Authors:** Yang Li, Jing Wang, Tao Sun, Xinlong Yu, Zhibo Yang, Yayun Zhao, Xuexi Tang, Hui Xiao

**Affiliations:** 1 College of Marine Life Sciences, Ocean University of China, Qingdao, 266003 China; 2 Laboratory for Marine Ecology and Environmental Science, Qingdao Marine Science and Technology Center, Qingdao, 266000 China

**Keywords:** *Sargassum thunbergii*, Endophytic bacteria, Community structure, Culture method, High-throughput sequencing

## Abstract

Endophytic bacteria are one of the symbiotic microbial groups closely related to host algae. However, less research on the endophytic bacteria of marine algae. In this study, the endophytic bacterial community of *Sargassum thunbergii* was investigated using the culture method and high-throughput sequencing. Thirty-nine endophytic bacterial strains, belonging to two phyla, five genera and sixteen species, were isolated, and *Firmicutes*, *Bacillus* and *Metabacillus indicus* were the dominant taxa at the phylum, genus and species level, respectively. High-throughput sequencing revealed 39 phyla and 574 genera of endophytic bacteria, and the dominant phylum was *Proteobacteria*, while the dominant genus was *Ralstonia*. The results also indicated that the endophytic bacteria of *S. thunbergii* included various groups with nitrogen fixation, salt tolerance, pollutant degradation, and antibacterial properties but also contained some pathogenic bacteria. Additionally, the endophytic bacterial community shared a large number of groups with the epiphytic bacteria and bacteria in the surrounding seawater, but the three groups of samples could be clustered separately. In conclusion, there are a variety of functional endophytic bacteria living in *S. thunbergii*, and the internal condition of algae is a selective factor for the formation of endophytic bacterial communities. This study enriched the database of endophytic bacteria in marine macroalgae, paving the way for further understanding of the interrelationships between endophytic bacteria, macroalgae, and the environment.

## Introduction

Endophytic bacteria are an important component of plant endophytic microorganisms and play an important role in the growth, development and disease resistance of host plants (Wang et al. 2015; Lu et al. 2022). The study of endophytic bacterial diversity not only provides valuable microbial resources, but also has important value in improving plant yield and elucidating the mechanisms of plant resistance and adaptability. Current research on plant endophytic bacteria is focused on higher terrestrial plants, especially agricultural crops, medicinal plants and environmental remediation plants. Numerous studies on the isolation, identification and determination of the physiological activity of endophytic bacteria from a wide range of terrestrial plants (Zhang et al. 2023; Purushotham et al. 2020) along with aquatic plant (Lan et al. 2008) and marine plants (Li et al. 2017; Zhang et al. 2021a; Ran et al. 2016) have been conducted since the middle of the 20th century.

Marine macroalgae, the main primary producers in coastal ecosystems, play an important ecological role by providing food, shelter, and habitat for many marine organisms, and having high industrial, agricultural, edible and medicinal value (Melo et al. 2021; Subramanian and Marudhamuthu 2020). However, only a few studies have also been carried out on the endophytic bacteria of marine macroalgae, mainly on some physiological activities of the isolated endophytic strains, such as the phosphorus-dissolving ability of the endophytic bacteria isolated from *Myriophyllum spicatum* (Guo et al. 2016), the resistance and tolerance of the endophytic bacteria of *Bacillus* and *Acinetobacter* to manganese stress, the growth-promoting ability of host plants isolated from wild and cultured *Myriophyllum verticillatum* (Zhang 2017), and the taxol-producing endophytic bacteria from *Sargassum polycystum* and *Acanthaphora specifera* (Subramanian and Marudhamuthu 2020), as well as the antibacterial activity of the endophytic bacteria isolated from *Acanthophora dendroides*, *Sargassum sabrepandum* (Ahmed et al. 2016) and *Eucheuma spinosum* (Sugrani et al. 2019), and the antioxidant activity of the endophytic bacteria isolated from *Ulva lactuca* L. and *Undaria pinnatifida* Suringar (Feng et al. 2020), etc.

Most of the above studies are based on the traditional cultivation method. High-throughput sequencing has been widely used to determine the endophytic bacterial communities of various higher plants, which can comprehensively clarify the structure and diversity of microbial communities. It is found that some dominant phyla are the same in the endophytic bacterial communities of different plants, but there are great differences at the genus level. For example, the three main phyla of the endophytic bacteria in sweet potato *Ipomoea batatas* (Puri et al. 2019) were found to be *Proteobacteria*, *Bacteroidetes* and *Actinomycetes*, similar to the dominant phyla of faba bean (*Vicia faba* L) seeds (*Bacteroidetes*, *Proteobacteria*, *Firmicutes* and *Actinomycetes*) (Liu et al. 2021b) and *Polygonatum cyrtonema* (*Proteobacteria*, *Actinomycetes*, *Gemmatimonadetes*, *Firmicutes* and *Acidobacteria*) (Cai et al. 2020). This is also very similar to the dominant endophytic bacteria in the aquatic plant *Spirodela polyrhiza* (*Proteobacteria*, *Bacteroides*, *Actinomycetes* and *Acidobacteria*) (Li et al. 2021). However, according to the results of high-throughput sequencing, the endophytic bacteria at the genus level vary greatly among species. For example, the core bacteria at the genus level in *Polygonatum* are composed of *Sphingomonas*, *Gemmatimonas* and *Streptomyces* (Cai et al. 2020). However, the endophytic bacteria at the genus level in *S*. *polyrhiza* are mainly some genera that are closely related to the biogeochemical cycles of nitrogen, carbon and phosphorus in water bodies, including *Pelomonas*, *Flavobacterium*, *Rubrivivax*, *Hydrogenophaga* and *Sphingomonas* (Li et al. 2021). Moreover, for the first time, many taxa have been reported as endophytic bacteria, such as the endophytic bacterial community found in rice growing in saline soil in West Bengal along with some previously unreported endophytic bacteria, such as *Aerinimonas*, *Arcobacter*, *Chitinophaga*, *Hydroispora* and *Sulfospirillum* (Kunda et al. 2018).

However, little is known about the structure of the endophytic bacterial communities in marine algae. only Hollants et al. (Hollants et al. 2011) have conducted a study about the endophytic bacterial communities in green seaweed *Bryopsis* (Bryopsidales, Chlorophyta) using the DGGE (denaturing gradient electrophoresis) method, and Mei analyzed the composition of endophytic bacterial community in* Sargassum horneri and Ulva prolifera * (Mei 2019) based the high-throughput sequencing. Additionally, Mangun et al. compared the isolated endophytic bacteria from healthy and diseased *Kappahycus alvarezii* (Mangun et al. 2023) Determining the composition and diversity of the endophytic bacterial communities in marine macroalgae using high-throughput sequencing technology is the first issue we want to investigate.

Additionally, endophytic bacteria in plants are closely related to their hosts and environment. Only a small proportion of endophytic bacteria in plants are unique to the plant and undergo vertical migration from parent to offspring through reproductive organs such as seeds or pollen. However, most of the bacteria in the whole plant are introduced from outside during plant growth (Wang et al. 2015). Many studies have shown that the composition and structure of epiphytic bacteria in marine macroalgae were different from those in surrounding waters (Mancuso et al. 2016; Pei et al. 2021), and it has also been found that the epiphytic and endophytic bacterial communities of *Gracilaria dura* were included in a sub-population of bacteria (Singh et al. 2015), However, few studies have simultaneously studied both endophytic and epiphytic bacterial communities and bacterial communities in the seawater surrounding macroalgae. The difference between the endophytic bacterial community in macroalgae, the epiphytic bacteria on the surface of macroalgae and the bacterial community in the seawater around macroalgae is another issue of interest to us.

*Sargassum thunbergii* is a common intertidal macroalgae along the northern coast of China, which has great value in medicine, the chemical industry, aquaculture and marine ecosystem restoration (Wu et al. 2010; Wang et al. 2022), and large-scale artificial cultures of this alga have been carried out. In this study, the community structure and diversity of the endophytic bacteria in *S. thunbergii* from the intertidal zone of Qingdao, Shandong Peninsula (China), were investigated using the culture method and high-throughput sequencing technology. At the same time, we compared their similarities and differences with the algal epiphytic bacteria and the bacterial community in the surrounding seawater. The purpose of our study was to broaden the basic information of marine algal endophytic bacterial communities and obtain culturable endophytic bacterial for further research. Our research helps to understand the relationship between algal endophytic bacteria, epiphytic bacteria and bacteria in the surrounding seawater and provides an experimental and theoretical basis for the development and utilization of *S. thunbergii*.

## Materials and methods

### Sample collection and processing

Well-grown *S. thunbergii* samples were collected from the rocky intertidal zone of Taipingjiao, Qingdao, Shandong Province (36°14’58.3"N, 120°21’34.2"E), in November 2020 and placed in sterile sample bags. The surrounding seawater was also collected in pre-sterilized sample bottles. Both the algal and seawater samples were stored in a portable ice box and transferred to the laboratory within 30 min for further processing.

### Sample surface disinfection

The algal samples were rinsed with sterile seawater to remove loosely adhering epiphytes and sand, and then the surface water of *S. thunbergii* was blotted out with sterile filter paper. In the pre-experiment, the samples were submerged in 75% ethanol for 5 min and in 2.5% sodium hypochlorite for 10 min successively, and then washed seven times with sterile double-distilled water (Liu et al. 2022). The final rinse was collected and incubated on Zobell 2216E agar medium plates at 25 °C in DNP-9082 Constant temperature incubator (Jinghong Experimental instrument Co., Ltd, Shanghai) for 72 h; when no bacterial growth was observed on the plate, we deemed the disinfection to be successful (Benjelloun et al. 2019; de Jong et al. 2019).

### Culture method

(1) Cultivation, isolation and preservation of endophytic bacteria.

An amount of 2.0 g of disinfected *S. thunbergii* was placed in a sterile mortar, adding 8 mL sterile physiological saline, which was then grinded to obtain a homogenous suspension. The initial suspension and decimal dilutions were used to isolate bacteria on Zobell 2216E agar medium plates. Three samples were treated, and the plates were incubated at 28 °C in DNP-9082 Constant temperature incubator (Jinghong Experimental instrument Co., Ltd, Shanghai) for 48 h. All colonies on a plate spread with one of the appropriate dilutions were isolated and purified several times, and then the obtained bacterial strains were preserved with cryoprotectant (0.9% physiological saline, 15% glycerol) (Peng et al. 2020) at -80 °C for further research.

(2) DNA extraction of endophytic bacteria.

Bacterial DNA was extracted using a bacterial genomic DNA extraction kit (TIANamp Bacteria DNA Kit), operated according to the kit instructions; only the second step was changed by adding 180 µL of 50 mg/mL lysozyme buffer with treatment at 37 °C for more than 30 min, while the other steps remained unchanged.

(3) Identification of endophytic bacteria via 16S rDNA sequencing.

After DNA extraction, sequencing was carried out by Sangon Biotech (Shanghai) Co., Ltd. The sequencing results were amplified using universal primers 27 F (AGAGTTTGATCCTGGCTCAG) and 1492R (TACGGYTACCTTGTTAYGACTT) from both ends using forward and reverse primers. The obtained sequences were spliced and compared in NCBI (www.ncbi.nlm.nih.gov) and Ezbiocloud (www.ezbiocloud.net) to determine the homology with known sequences, and those with more than 97% sequence similarity were classified as the same species.

(4) Phylogenetic analysis.

The software MEGA 7.0 was used to compare the sequences of the strains with those of similar strains from Ezbiocloud (www.ezbiocloud.net), and the matrix distance was calculated from the sequence data according to the “Kimura two-parameter” method. The neighbor-joining method was used to perform 1000-replicate validation and phylogenetic tree analysis.

### High-throughput sequencing

#### Sample processing

The endophytic bacteria, epiphytic bacteria and bacteria in the surrounding seawater were named AS, ASE and AW, respectively, and each group had three replicates. Each replicate was from one sampling site.

(1) Endophytic bacteria.

An amount of 2.0 g of disinfected *S. thunbergii* was placed in lyophilization tubes and stored at -80 °C until DNA extraction.

(2) Epiphytic bacteria.

Epiphytic bacteria samples were obtained in accordance with the previous literature (Mathai et al. 2018). Approximately 25 g of each sample of *S. thunbergii* was transferred to a 250 mL conical flask, followed by the addition of 70 mL of 0.01 M phosphate buffer (pH 7.4), and then the flask was sealed with sterilized film and shaken for 30 min at room temperature in a rotary HZQ-C shaker (HDL, Harbin, China) at 200 r min^-1^ to obtain a suspension of epiphytic bacteria isolated from the surface of the algae. The suspension of epiphytic bacteria was filtered through a sterile 500-mesh sieve to remove impurities such as sand mixed in the suspension. The bacteria were collected onto sterile 0.22 μm filter membranes (Millipore, Merck KGaA, Darmstadt, Germany) via filtration in a sterile environment using vacuum filtration system (Steriflip, Millipore, USA), and the membranes were stored at -80 °C until DNA extraction.

(3) Bacteria in the surrounding seawater.

The bacteria in the surrounding seawater (500 mL) were collected onto 0.22 μm membranes via vacuum filtration in a sterile environment and stored at -80 °C until being processed for DNA extraction within a week.

### High-throughput sequencing and sequence processing

High-throughput sequencing was conducted by Guangzhou Genedenovo Biotechnology Co., Ltd (Guangzhou, China). The genomic DNA was extracted from the samples, and the V3 + V4 regions of 16S rDNA were amplified with specific primers 341 F (CCTACGGGNGGCWGCAG) and 806R (GGACTACHVGGGTATCTAAT). The PCR amplification products were recovered via gel splicing and quantified using a Quanti Fluor ^TM^ fluorometer (Promega, United States). Equal amounts of purified amplification products were mixed and sequencing adapters were attached; then, sequencing libraries were constructed using an Illumina Hiseq2500 PE250 (Illumina, Inc., San Diego, CA, USA).

### Data analysis

Sequences were spliced and de-duplicated using Uparse software (v9.2.64_i86linux32) (Edgar 2013), and species with > 97% similarity were clustered into operational taxonomic units (OTU). Chloroplast and mitochondrial sequences were removed from the OTU tables. The obtained OTUs were classified based on the Greengenes database (version gg_13_5, greengenes.secondgenome.com/) and Bacterial SILVA database (SILVA version 106, http://www.arb-silva.de/documentation/release-106/) databases (Janssen et al. 2018), and the pre-processing of data and the selection of OTUs from the amplicons were performed using Mothur pipelines (version 1.39.1 available online: https://mothur.org/wiki/miseq_sop/mothur) (Schloss et al. 2009) to calculate the diversity indices, namely Shannon, Simpson, Ace, the Chao1 index and coverage; β-diversity was calculated and analyzed via unweighted UniFrac distances for principal coordinate analysis (PCoA) and the unweighted pair group method with arithmetic mean (UPGMA). The resulting high-throughput sequencing data were clustered according to OTU classification for species diversity at the phylum and genus levels. Species without a clear classification and species with a relative abundance of less than 1% were jointly recorded as Other, and histograms were drawn. Linear discriminant analysis effect size (LEfSe) software (v1.0, https://huttenhower.sph.harvard.edu/galaxy/) was used to analyze the differences between groups (Qin et al. 2020). In LEfSe, the Kruskal–Wallis rank sum test was first performed among all groups of samples, and the screened differences were then compared between two groups using the Wilcoxon rank sum test. The results were ranked using linear discriminant analysis (LDA) to obtain the LDA variance analysis graph, and then the evolutionary branching graph was obtained by mapping the differences to a classification tree with a known hierarchical structure.

## Results

### Isolation and identification of culturable bacteria

**Fig. 1:**
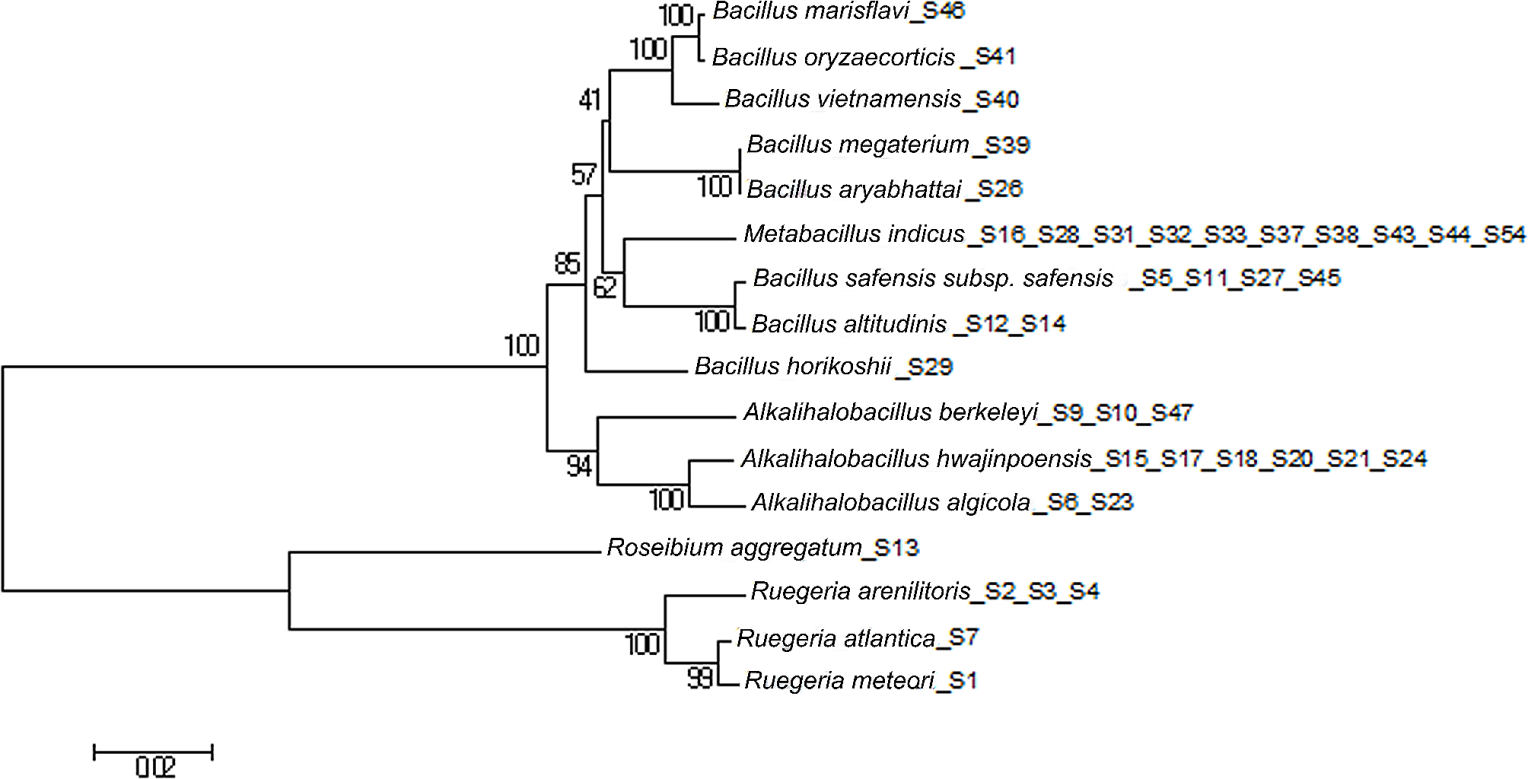
Phylogenetic tree of culturable endophytic bacteria in *S. thunbergii* based on 16S rDNA using neighbor-joining methods. Associated taxa were clustered in the bootstrap test (1000 replicates), and the bootstrap values were greater than 50%

A total of 54 strains of endophytic bacteria were isolated from *S. thunbergii*, but only 39 strains were finally identified because some strains preserved in sterilized 0.9% physiological saline containing 15% glycerol at -80℃ were not successfully activated. A phylogenetic evolutionary tree based on the molecular biological identification of the strains is shown in Fig. 1. These strains belong to 2 phyla, 5 genera and 16 species (Table 1). At the phylum level, *Firmicutes* was overwhelmingly dominant (85.00%), including the three genera *Bacillus*, *Alkalihalobacillus* and *Metabacillus*; the other phylum was *Proteobacteria* (15.00%), including the two genera *Ruegeria* and *Roseibium*. At the genus level, the dominant genera were *Bacillus* (31.00%), *Alkalihalobacillus* (28.30%) and *Metabacillus* (25.70%); at the species level, the dominant species were *M. indicus* (25.70%), *A. hwajinpoensis* (15.40%) and *B. safensis* subsp. *safensis* (10.30%).

**Table 1:** Identification results of culturable endophytic bacteria in *S. thunbergii*

Phylum	Genus	Species	Number	Proportion (%)
*Firmicutes*	*Bacillus*	*Bacillus safensis* subsp. *safensis*	4	10.30
		*Bacillus horikoshii*	1	2.60
		*Bacillus altitudinis*	2	5.10
		*Bacillus vietnamensis*	1	2.60
		*Bacillus oryzaecorticis*	1	2.60
		*Bacillus megaterium*	1	2.60
		*Bacillus marisflavi*	1	2.60
		*Bacillus aryabhattai*	1	2.60
	*Alkalihalobacillus*	*Alkalihalobacillus hwajinpoensis*	6	15.40
		*Alkalihalobacillus algicola*	2	5.10
		*Alkalihalobacillus berkeleyi*	3	7.80
	*Metabacillus*	*Metabacillus indicus*	10	25.70
*Proteobacteria*	*Ruegeria*	*Ruegeria atlantica*	1	2.60
		*Ruegeria meteori*	1	2.60
		*Ruegeria arenilitoris*	3	7.80
	*Roseibium*	*Roseibium aggregatum*	1	2.60
**Sum**			**39**	

### Results of high-throughput sequencing

#### Biodiversity

##### α-Diversity

**Fig. 2:**
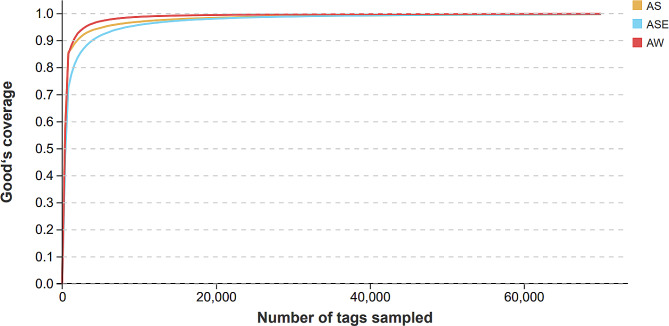
Coverage dilution curve of the endophytic and epiphytic bacterial communities of *S. thunbergii* and bacterial community in seawater samples

The results of the high-throughput sequencing of the endophytic and epiphytic bacteria of *S. thunbergii* and bacteria in the surrounding seawater samples were compared and analyzed, and a total of 1,062,077 sequences were obtained. A total of 1,011,972 optimized sequences were obtained after quality filtering and removal of chimera, chloroplast and mitochondrion sequences, and the coverage of each sample was above 99%, as shown in Fig. 2, indicating that the sequencing depth covered most of the bacteria in the samples, and that the sequencing data are reliable and appropriate.

The analysis results of the Chao1 index and ACE index (Table 2) showed that the richness of the endophytic bacteria was in the middle—lower than that of the epiphytic bacteria and higher than that of the seawater samples—but the Shannon index and Simpson index showed that the diversity of the endophytic bacteria was lower than that of the epiphytic bacteria and bacteria in the surrounding seawater. It is worth mentioning that the four diversity indices of the epiphytic bacterial community were higher than those of the other two groups of samples.

**Table 2:** The α-diversity of the endophytic and epiphytic bacterial communities of *S. thunbergii* and bacterial community in seawater samples

Sample	Shannon	Simpson	Chao	Ace
Endophytic bacteria	5.84	0.87	1612.15	1627.61
Epiphytic bacteria	8.67	0.99	2142.81	2137.17
Bacteria of surrounding seawater	7.3	0.98	1127.63	1160.46

##### β-Diversity

**Fig. 3:**
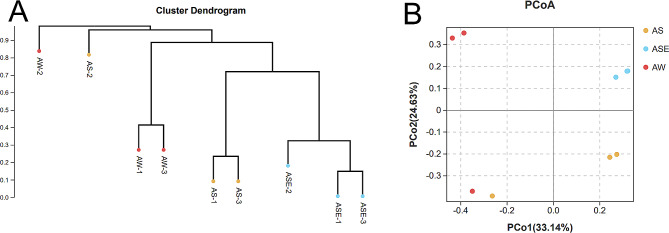
The β-diversity of the endophytic and epiphytic bacterial communities of *S. thunbergii* and bacterial community in seawater samples (A: UPGMA results; B: PCoA analysis results)

Figure 3 shows the results of the Bray-based UPGMA and PCoA analysis. The clustering analysis showed that the compositions of the endophytic and epiphytic bacterial communities of *S. thunbergii* and bacterial community in the seawater samples were similar within the groups, and only one sample of endophytic bacteria differed obviously from the other two samples in the group, being similar to the bacterial samples from the seawater. The results of the PCoA analysis showed that the structure of the bacterial communities differed significantly between groups (*P* < 0.05, ANOSIM test), indicating that the endophytic bacterial communities were different from the bacterial communities of the epiphytic bacteria and seawater samples.

##### Composition of the bacterial community

**Fig. 4:**
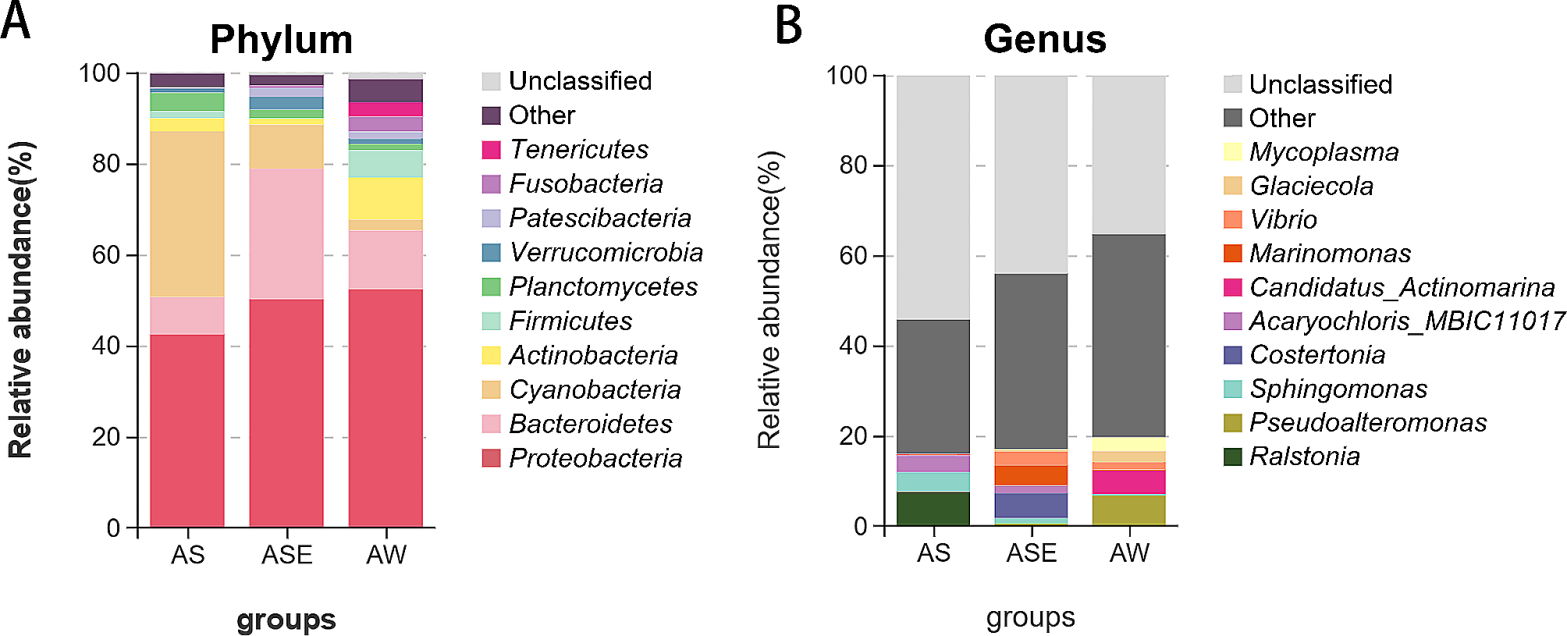
Relative abundance of endophytic and epiphytic bacterial communities of *S. thunbergii* and bacterial community in seawater samples (A: phylum level; B: genus level)

The relative abundance of the endophytic bacterial community structure of *S. thunbergii* at the phylum and genus levels is shown in Fig. 4A. At the phylum level, the most dominant phylum of the endophytic bacteria was *Proteobacteria* (42.47%), followed by *Cyanobacteria* (36.41%), *Bacteroides* (8.16%), *Planctomycetes* (4.03%) and *Actinomycetes* (2.84%). The dominant phyla of the epiphytic bacteria were similar to those of the endophytic bacteria and bacteria in the seawater samples, with the most dominant phylum being *Proteobacteria*, but its abundance was lower than that in the other two groups of samples (50.15% and 52.37%, respectively). Similarly, *Bacteroides*, as the third most dominant phylum of the endophytic bacteria, was the second most dominant phylum in the epiphytic bacteria and seawater samples, with abundances of 28.68% and 12.95%, respectively, both of which were higher than the abundance in the endophytic bacteria. However, the abundance of *Cyanobacteria* in the endophytic bacteria (36.41%) was significantly higher than that in the epiphytic bacteria (9.68%) and seawater samples (2.34%). The abundances of *Actinomycetes* and *Firmicutes* in the endophytic bacteria were 2.84% and 1.59%, respectively—in between those of the epiphytic bacteria (1.27%/0.11%) and seawater samples (9.23%/5.97%).

At the genus level (Fig. 4B), the most dominant genus of the endophytic bacteria was *Ralstonia* (7.53%), followed by *Sphingomonas* (4.36%), *Acaryochloris_MBIC11017* (3.65%). The dominant genera in the epiphytic bacteria and seawater samples were completely different, with the most dominant genus of the epiphytic bacteria being *Costertonia* (5.58%), followed by *Marinomonas* (4.47%), *Vibrio* (3.02%), *Acaryochloris_MBIC11017* (1.71%) and *Sphingomonas* (1.21%), while the most dominant bacteria in the seawater samples were *Pseudoalteromonas* (6.68%), followed by *Candidatus_Actinomarina*, (5.07%), *Mycoplasma* (3.05%), *Glaciecola* (2.49%) and *Vibrio* (1.65%).

**Fig. 5:**
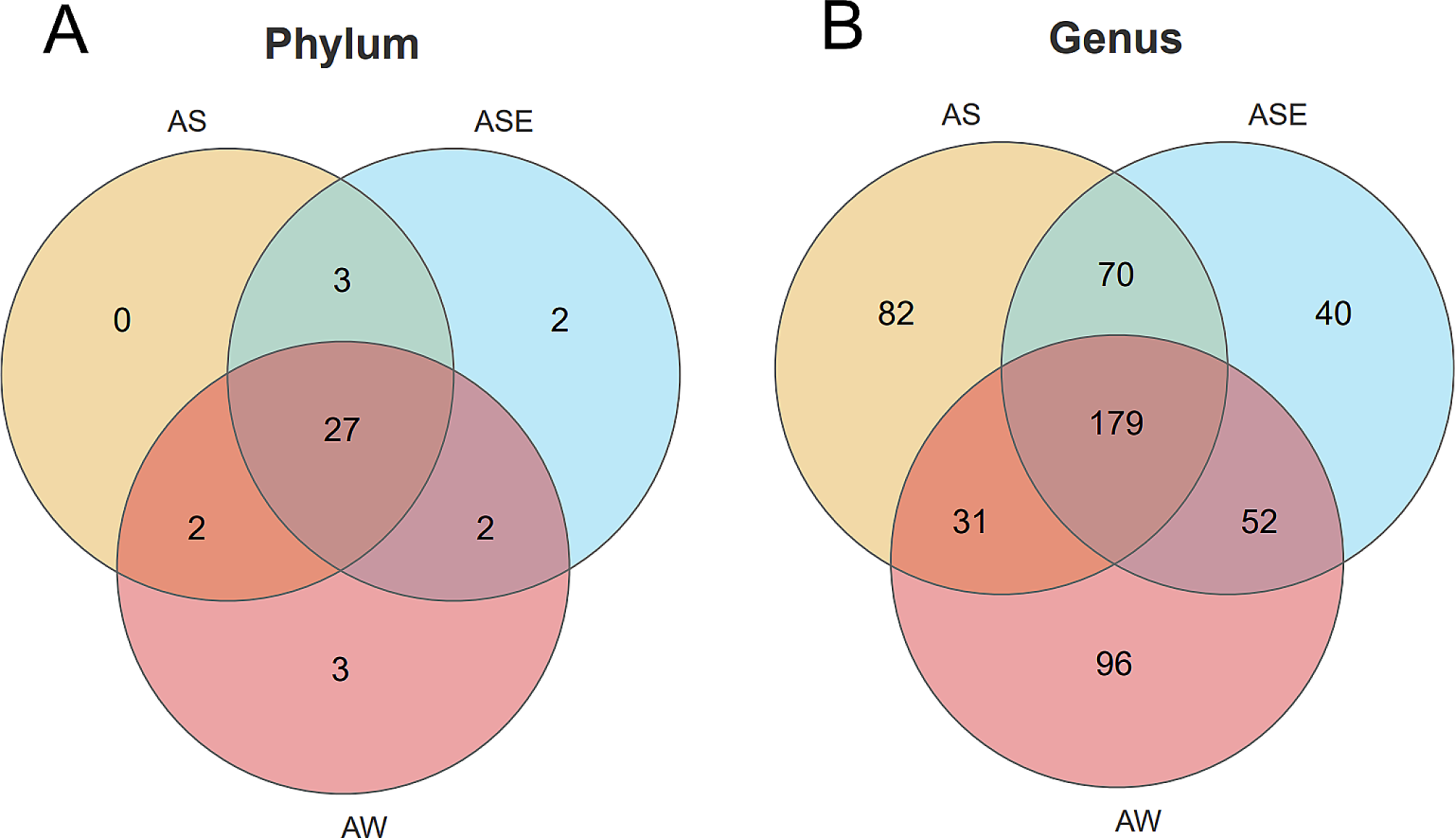
Differences between endophytic and epiphytic bacteria of *S. thunbergii* and bacteria in seawater samples at the phylum and genus levels (A: phylum level; B: genus level)

As can be seen in the Venn diagram (Fig. 5A), 27 phyla were shared by the endophytic bacteria, epiphytic bacteria and seawater samples. There was no specific phylum in the endophytic bacteria, but the specific phyla in the epiphytic bacteria were *Cloacimonetes* and *Rokubacteria*; the specific phyla in the seawater samples were *Latescibacteria* and *Synergistetes*, indicating that the richness of the endophytic bacteria at the phylum level was lower than that of the epiphytic bacteria and seawater samples.

At the genus level (Fig. 5B), 179 genera were shared by the endophytic bacteria, epiphytic bacteria and seawater samples. Among the shared bacteria, the highest abundance among the endophytic bacteria was found for *Acaryochloris_MBIC11017*, *Sphingomonas* and *Sphingobium*, and the highest abundance among the epiphytic bacteria was found for *Costertonia*, *Marinomonas* and *Vibrio*, while the most abundant bacteria in the seawater samples were *Pseudoalteromonas*, *Candidatus_Actinomarina* and *Glaciecola*. In addition, there were 82 specific genera in the endophytic bacteria, mainly including *Hydrogenophaga*, *Mesorhizobium*, *Methyloversatilis*, *Weissella*, *Fimbriiglobus*, *Nevskia* and *Pseudoxanthomona*, *Arsenophonus*, *Buchnera*; 40 specific genera in the epiphytic bacteria, mainly including *Epixenosomes_of_Euplotidium_arenarium*, *Cupriavidus* and *Amphiplicatus*; and 96 specific genera in the seawater samples, mainly including *Capnocytophaga*, *Providencia*, *Rothia* and *Fusobacterium*.

##### Indicator species

**Fig. 6:**
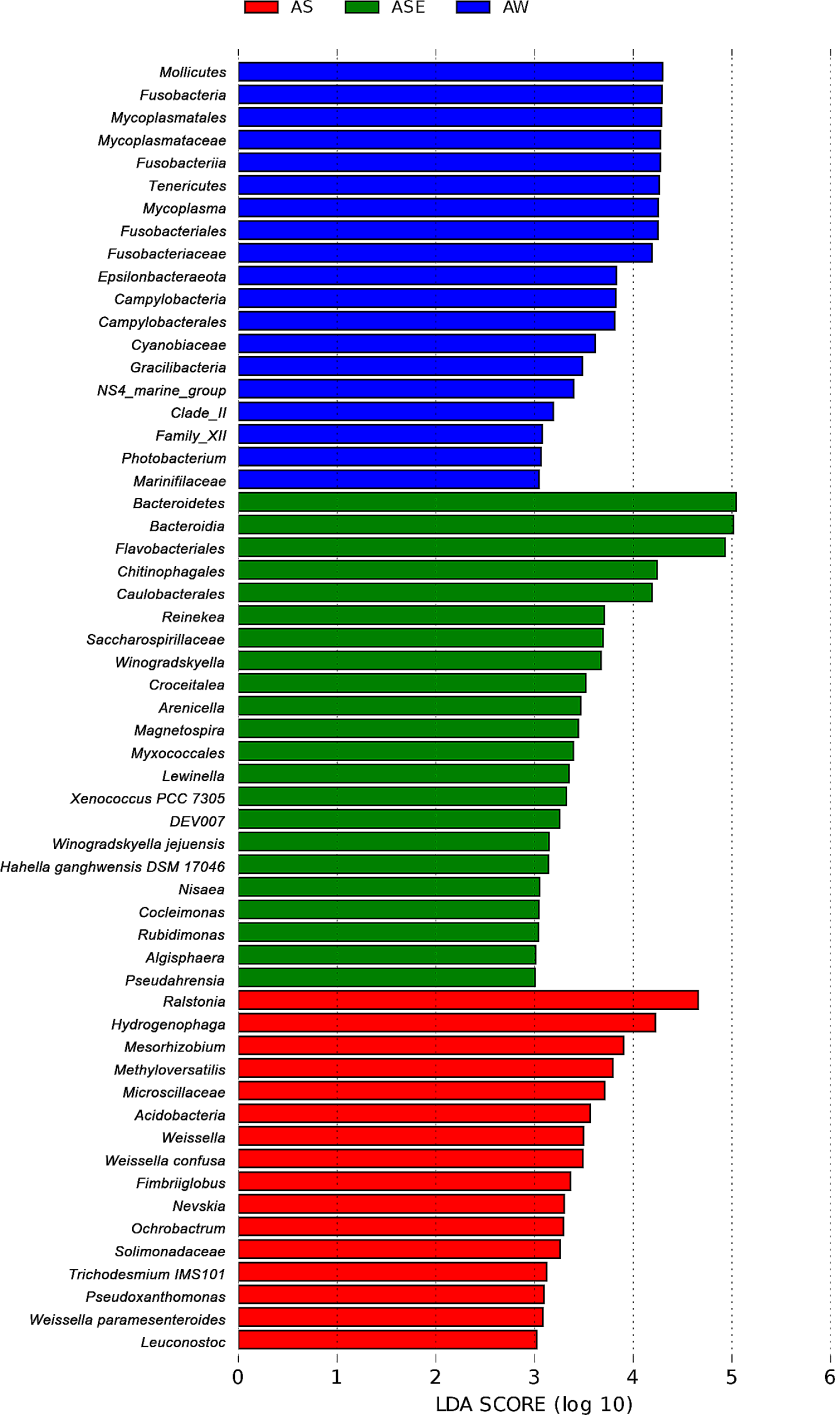
Indicator taxa of the endophytic and epiphytic bacterial communities of *S. thunbergii* and bacterial community in seawater samples. Different colors indicate different groups, and LDA score (effect size) indicating significant differences in bacterial taxa (LDA score > 3.0)

LEfSe was used to identify bacterial taxa with significant differences in abundance between groups, i.e. indicator species (Dufrêne and Legendre, 1997) and the results (Fig. 6) showed that the indicator species of endophytes were not the same as those of the epiphytic bacteria and seawater samples. The indicator taxa of the endophytic bacteria were mainly *Ralstonia* (genus), *Hydrogenophaga* (genus) and *Mesorhizobium* (genus), while *Bacteroidetes* (phylum), *Bacteroidia* (class) and *Flavobacteriales* (order) were enriched in the epiphytic bacteria, and *Mollicutes* (class), *Fusobacteria* (phylum) and *Mycoplasmatales* (order) were abundant in the seawater samples.

**Fig. 7:**
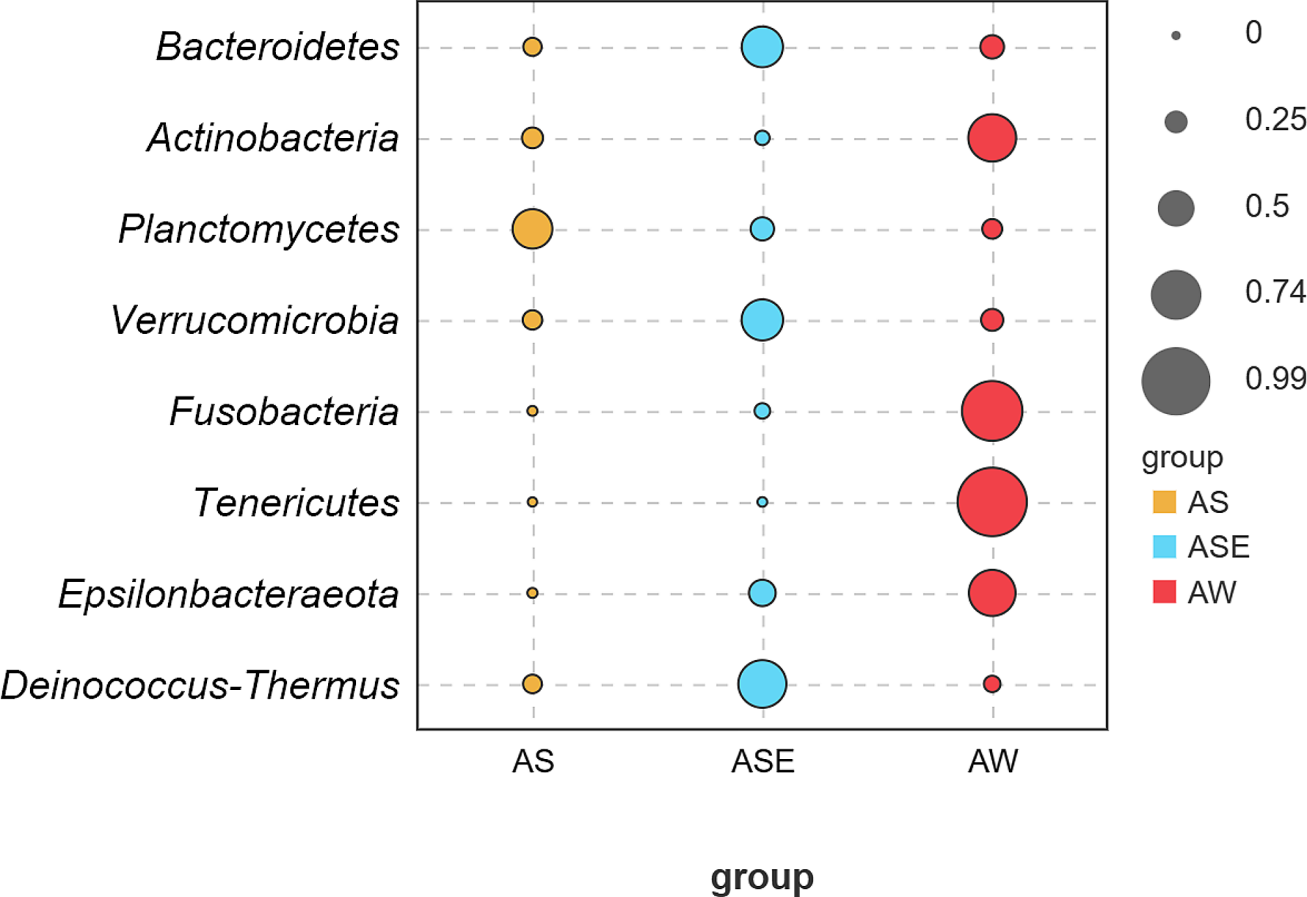
Indicator value of endophyte bacteria, epiphytic bacteria of *S. thunbergii* and bacteria of seawater samples

Figure 7 showed the bacteria with significant differences in abundance at phylum level between endophytic, epiphytic bacteria of *S. thunbergii* and seawater samples at the phylum level (*P* < 0.05). Only *Planctomycetes* had the largest indicator value in endophytic bacteria while *Bacteroidetes, Verrucomicrobia* and *Deinococcus-Thermus* were enriched in epiphytic bacteria. On the contrary, these phyla with high indicator values in endophytic or epiphytic bacteria had low indicator values in seawater samples, while *Actinobacteria*, *Fusobacteria*, *Tenericutes* and *Epsilonbacteraeota* with low abundance in endophytic or epiphytic bacteria were enriched in seawater samples.

## Discussion

In this study, the community structure of the endophytic bacteria in *S. thunbergii* was investigated using a combination of the traditional culture method and high-throughput sequencing technology, the bacterial community structure of the epiphytic bacteria and bacteria in seawater samples was also analyzed. The results showed that the endophytic bacterial communities of *S. thunbergii* had some dominant phyla in common with those of other marine macroalgae and terrestrial plants, but the dominant genera were very different.

The dominant phyla of the endophytic bacteria of *S. thunbergii* obtained using the culture method were *Firmicutes* and *Proteobacteria* and these two phyla were also dominant in *K. alvarezii* (Mangun et al. 2023). This result was also consistent with the previous studies showing that the dominant phylum of the endophytic bacteria of terrestrial plants was *Proteobacteria*, followed by a small number of *Firmicutes*, *Actinomycetes* and *Bacteroides* (Trivedi et al. 2020; Edwards et al. 2015). However, the dominant genus and species of the endophytic bacteria in *S. thunbergii* were different from those in *K. alvarezii* (Mangun et al. 2023). *Alkalihalobacillus*, *Metabacillus*, *Ruegeria* and *Roseibium* were reported as plant endophytes for the first time, but these bacteria are common in marine environments or marine organisms. For example, *Alkalihalobacillus* is a salt-tolerant bacterium (Li et al. 2021); *Metabacillus* has been reported to induce coral larval sedimentation and metamorphosis (Zhang et al. 2021b); *Ruegeria* is common in cultured waters, having been reported to be present in the intestine of *Anguilla rostrata* (Liu et al. 2021a); *Roseibium* spp. are the dominant intestinal species in healthy *Hippocampus* (Yan et al. 2020). At the species level, the endophytic nitrogen-fixing bacterium *M. indicus* was the most abundant, indicating that endophytic bacteria may play a certain role in the acquisition of nitrogen in algae, followed by *A. hwajinpoensis*, which is a salt-tolerant strain with a denitrification function (Cao et al. 2019). The third most dominant bacterium was *B. safensis* subsp. *safensis*, which has been reported many times in terrestrial plants and is a biocontrol endophyte with a wide range of antibacterial activities against a variety of pathogenic fungi and bacteria (Zhang et al. 2020b).

The results of the high-throughput sequencing revealed that the dominant phyla of the endophytic bacteria of *S. thunbergii* were *Proteobacteria*, *Cyanobacteria*, *Bacteroides*, *Planctomycetes* and *Actinomycetes* This result was similar to that of the dominant phylum of endophytic bacteria in *S. horneri* and *U. prolifera* (Mei 2019), but it varied considerably at the genus level, without the same dominant genera. The most dominant genus of the endophytic bacteria was *Ralstonia*. *Ralstonia solanacearum*, the pathogen of a devastating worldwide crop bacterial disease, is a member of this genus (She and He 2020). Other dominant genera include important degraders in the environment (Liu et al. 2017), such as *Sphingomonas*, *Hydrogenophaga* and *Sphingobium*and *Cyanobacteria, Acaryochloris_MBIC11017*. Further analysis of other genera of endophytic bacteria revealed that the most abundant taxa were bacteria with degradation functions, including the highly efficient benazolin-ethyl degrading *Methyloversatilis* (Qian et al. 2011), chitinolytic *Fimbriiglobus* (Ravin et al. 2018), pyrene-degrading *Pseudoxanthomonas* (Klankeo et al. 2009), cellulolytic *Ferrovibrio* (Dahal et al., 2018), denitrifying *Janthinobacterium* (Yang 2019), organic-matter-degrading *Lysinibacillus* (Li et al. 2018; Zhu 2015), members of *Pseudorhodoplanes* (Zhen et al. 2019), which degrade complex organic matter, and members of *Phenylobacterium*, comprising the best indigenous petroleum-hydrocarbon-degrading bacteria, suggesting that endophytic bacteria have various strong degradation functions. In addition, there were also multiple nitrogen-fixing bacteria, such as *Mesorhizobium* (Yang 2017) and some members of *Cyanobacteria*, including *Arthrospira_PCC-7345* (Du et al. 2016), *Trichodesmium_IMS101* (Kranz et al. 2010), *Rhodobacter* (Jin et al. 2019) and *Niveispirillum* (Cai et al. 2018). There were also some bacteria with antibacterial activity, or bacteria that are generally considered beneficial, such as the new antibacterial agent and probiotic *Weissella*, (Tenea and Hurtado 2021), *Arsenophonus* which contributes to the resistance of *Nilqparvata lugens* (Chen et al. 2014), *Buchnera* (Li and Li 2006), whose members are specialized symbiotic bacteria that provide a variety of essential amino acids and B vitamins to the host aphid, and intestinal beneficial bacteria belonging to *Bifidobacterium* (Li and Zhu 2022). It was interesting that there are also some pathogenic bacteria among the endophytic bacteria, including the most dominant genus *Ralstonia* comprises pathogens that causes plant bacterial wilt and fish pathogen *Plesiomonas* (Chen et al. 2020), *Neorickettsia*, *Legionella* and *Acidovorax* (Zhang et al. 2019). In summary, the composition and function of the endophytic bacterial community of *S. thunbergii* are very complicated. How these bacteria interact with the host *S. thunbergii* and finally form a stable symbiosis within *S. thunbergii* needs to be further investigated.

In addition, the community composition of the endophytic bacteria of *S. thunbergii* were significantly different compared to those of the epiphytic bacteria and bacteria in the seawater samples. Although 27 phyla and 179 genera were shared, the 3 groups of samples could be clustered separately in the β-diversity analysis, indicating the variability between the bacterial communities from the three types of samples. Firstly, the relative abundance of the dominant phyla differed significantly. The abundance of *Cyanobacteria* was absolutely dominant, far exceeding the abundance in the epiphytic bacteria and in the seawater samples, indicating that the symbiotic cyanobacteria of *S. thunbergii* mainly live inside the algal body. The abundance at the genus level also varied greatly, and some genera were highly abundant inside the algae, such as *Acaryochloris_MBIC11017*, which was obviously higher in the endophytic bacteria than in the epiphytic bacteria and seawater samples; this was consistent with the result that the phylum *Cyanobacteria* was dominant in the endophytic bacteria, indicating that a large number of *Cyanobacteria* live inside of *S. thunbergii*. There were also some bacteria with a low abundance in the endophytic bacteria, such as the marine oil-degrading *Pseudoalteromon* (Zhang et al. 2020a), with an obviously lower abundance in the endophytic bacteria than in the epiphytic bacteria and the seawater samples. This suggests that the taxa of endophytic bacteria and their functions differ significantly from those of bacteria on the surface of macroalgae and in the surrounding seawater. In addition, some pathogenic bacteria were also found in extremely low abundance inside the seaweed, such as *Mycoplasma*, whose abundance inside the macroalgae was much lower than in the epiphytic bacteria and seawater samples. This was also the case for *Vibrio*. The low abundance of these pathogen in the endophytic bacteria of *S. thunbergia* suggests that the cell wall of *S. thunbergii* is resistant to the invasion of pathogenic bacteria.

The endophytic bacteria also differed from the epiphytic bacteria and bacterial communities in the seawater samples in the indicator species and specific bacteria of each group. For example, the indicator species *Ralstonia* was highly abundant in endophytic communities; however, it was not found in the epiphytic bacteria, and it only had an abundance of 0.02% in the seawater samples. *Ralstonia*, includes *Ralstonia solanacearum*, an important pathogen that causes wilt disease in many land plants (She and He 2020), as well as many strains of organic pollutants-degrading bacteria (Coll et al. 2020) and phosphate-solubilizing (Dandessa and Bacha 2018). *Ralstonia* was also found in plant seeds (Liu et al. 2011), indicating that *Ralstonia* can be transmitted from parents to offspring without the need to enter the plant body from the external environment. This may be the reason why *Ralstonia* was not present in the epiphytic bacteria in *S. thunbergii* in this study, while its abundance was high in the endophytic bacteria.

Similarly, the degrading bacterium *Hydrogenophaga* (Liu et al. 2017) was enriched in the endophytic bacteria, but this genus was absent in both the epiphytic bacteria and bacteria in the seawater samples, suggesting that not all endophytic bacteria in *S. thunbergii* enter from the surrounding seawater. In contrast, the human pathogenic bacterium *Capnocytophaga* (Ye et al. 2018) was enriched in the seawater samples, but it was absent in both the endophytic and epiphytic bacteria, suggesting that it selectively enters *S. thunbergii* and forms a community different from that in the surrounding seawater and on the algal surface.

Both the culture method and high-throughput sequencing technology indicated that there were abundant endophytic bacterial groups in *S. thunbergii*. Moreover, the number of bacterial taxa obtained using high-throughput sequencing technology was much higher than that obtained using the culture method, and the dominant bacterial taxa obtained using these two methods were different. This may be mainly due to the limitations of the culture method, where only approximately 1% of the bacteria found in the natural environment are cultivable (Eilers et al. 2000). Endophytic bacteria inhabit the interiors of plants, whose community composition is closely related to the internal physiological and biochemical conditions of host plants. Due to the fact that during in vitro cultivation, the bacterial culture medium does not contain algae-derived substances, many groups of bacteria cannot be cultivated (Mitrani et al. 2005). Further, the commonly used method of isolating endophytic bacteria from plants actually isolates aerobic bacteria. Although the algal cell walls are permeable, the results of the high-throughput sequencing in this study revealed that the endophytic bacteria include anaerobic bacteria such as *Hydrogenophaga* (Tan et al. 2021) and *Nevskia* (Li 2017), which cannot grow under aerobic conditions. Therefore, the taxa of the endophytic bacteria isolated using the culture method in this study were fewer in number than those obtained using high-throughput sequencing, but the culture method can isolate and obtain a large number of purified strains for further studies of bacteria–algae interactions, as well as the development of functional strains. Therefore, it is still important in the study of endophytic bacteria in algae.

In conclusion, the composition of the endophytic bacterial community of *S. thunbergii* was studied using the culture method and high-throughput sequencing technology in this study. It was found that the endophytic bacteria in *S. thunbergii* are abundant and enriched in groups with nitrogen fixation, salt tolerance, pollutant degradation, and antibacterial properties as well as pathogenic bacteria. In addition, the communities of the endophytic bacteria differed from that of epiphytic bacteria in *S. thunbergii* and the bacteria in the surrounding seawater. This study provides a preliminary understanding of the structure of the endophytic bacterial community of *S. thunbergii* and helps to elucidate the mechanism of bacterial–algal relationships. Moreover, a large number of endophytic bacterial strains were obtained, providing experimental materials for the effective utilization and development of bacterial resources.

## Data Availability

All bacterial 16S rRNA gene sequence data produced during the study were deposited in the NCBI Sequence Read Archive database under Bioproject PRJNA835680. Results for concurrent bacterial cultures are available in the NCBI SRA repository under the BioProject ID: PRJNA841409.
